# Improving preschool nutrition: addressing knowledge and practice gaps among nursery staff in Amman, Jordan

**DOI:** 10.3389/fnut.2025.1621609

**Published:** 2025-09-30

**Authors:** Hadieh Al-Mahshi, Areen Haddad, Ruba Musharbash, Haitham Jahrami, Adam Amawi, Hadeel Ghazzawi

**Affiliations:** ^1^Department of Nutrition and Food Technology, School of Agriculture, The University of Jordan, Amman, Jordan; ^2^Dr. Ruba Musharbash Center for Nutrition Counseling, Amman, Jordan; ^3^Department of Psychiatry, College of Medicine and Medical, Sciences, Arabian Gulf University, Manama, Bahrain; ^4^Department of Exercise Sciences and Kinesiology,School of Sport Sciences, The University of Jordan, Amman, Jordan

**Keywords:** preschoolers, nurseries, knowledge, nutrition, teacher

## Abstract

**Background:**

Preschoolers attending nurseries typically spend a significant period of their day in nurseries, where they consume approximately half or more of their daily meals under the supervision of nursery staff. As the primary caregivers during this time, nursery staff play a vital role in forming children’s eating habits and influencing their nutritional intake. Thus, assessing the nutritional knowledge and practices of nursery staff is essential.

**Objective:**

To assess the nutritional knowledge and practices of nursery staff members.

**Methods:**

A cross-sectional study was conducted among nursery staff. A self-administered questionnaire was used to assess the nutritional knowledge and practices of the nursery staff members.

**Results:**

A total of 53.2% of the nursery staff exhibited high levels of nutritional knowledge, whereas 3.2% showed low nutritional understanding. Additionally, approximately 50% of the nursery staff displayed excellent dietary behaviors, while 6.4% showed inadequate standards.

**Conclusion:**

Nursery staff possess preliminary nutrition knowledge but need guidance and training sessions in nutrition that may enhance their essential role in improving preschoolers’ nutritional status and health.

## Introduction

Teachers significantly influence a child’s life, as their knowledge (what they know about nutrition), attitudes (their beliefs and feelings toward nutrition), and practices (their actual behaviors and actions) play a crucial role in shaping children’s nutritional and health habits. These components are often interrelated and are essential in designing effective health education interventions (1). Numerous studies have investigated the impact of childcare centers on preschooler’s nutrition by employing questionnaires to measure caregivers’ knowledge, attitudes, and nutritional practices (2–5). In contrast, other studies have centered on the efficacy of nutrition education interventions for children, such as a review study highlighting interventions aimed at promoting fruit and vegetable consumption (6). Additionally, research by Motebejana et al. (7), highlighted the importance of establishing recommendations and guidelines to assist caregivers of preschool children under 5 years in understanding their nutritional and dietary needs and enhancing their nutrition status.

Teachers and parents both play essential roles in encouraging healthy dietary practices among children, with nurseries serving as a vital environment for family and child development by providing opportunities to promote positive eating habits and nutritional education (8, 9). Nutritional knowledge and education often start in early childhood (10). And when this subject is consistently paired with improved meal practices, it assists in the formation and enhancement of healthy eating patterns in children. By the age of four, children begin to understand the connection between health and nutrition (11); nevertheless, this understanding does not always result in healthy food choices, particularly among young children (12).

Possessing nutritional knowledge alone does not always guarantee its effective application in daily activities. Hence, the successful implementation of nutrition for children requires careful planning guided by specialized protocols (13). According to regional dietary guidelines for preschool-aged children (2–5 years), the recommended daily energy intake ranges between 1,200 and 1,600 kcal, with macronutrient distribution comprising approximately 45–65% carbohydrates, 10–15% protein, and 30–40% fat (14). Globally, many childcare centers aim to provide at least 1/3 to 1/2 of the recommended daily intake during childcare hours (15).

Preschoolers often consume between three or four meals during their time at nursery, covering more than half of their daily nutritional needs. As a result, failure to adopt fundamental nutrition practices and adhering to improper eating habits is intolerable in this setting, as it may result in adverse health consequences in childhood or later stages. Therefore, the requirement of ongoing education of the nursery staff, including reinforcement, awareness-raising, and encouraging daily healthy nutritional practices is a vital need (16).

The childcare environment fosters a strong connection between the caregiver and the child, positioning caregivers as key role models whose behavior children tend to observe and imitate. This interaction plays a significant role in shaping children’s nutritional habits and foundational understanding of health. Moreover, childcare settings provide an ideal platform for large-scale educational programs because the habits formed there interact with and reinforce those learned at home, creating a complementary effect on children’s overall development (17).

## Methods

### Study design

This cross-sectional study was carried out among 62 nurseries’ staff from 20 nurseries distributed in all regions of Amman to evaluate the nutritional knowledge and practices of nurseries staff’ in Amman, Jordan. Each participating daycare facility typically employed five to seven staff people, including caregivers and administrative staff, and catered to 20 to 30 preschool-aged children. In addition to reflecting average nursery conditions in the area, this staff-to-child ratio offers pertinent background for interpreting the study’s assessment of knowledge and practice levels. However, based on logistical feasibility and the average number of nursery workers available per facility, 62 participants were chosen as the sample size. Although a formal sample size calculation was not carried out, the chosen sample size was thought to be sufficient to offer preliminary insights into the nutritional practices and knowledge of Amman nurseries’ employees.

### Sampling procedure

The Capital Governorate, Amman, is divided into five regions: North, South, Center, East, and West, according to the Department of Lands and Survey. Cluster sampling was applied to select the nurseries in this study depending on the density of nurseries in the region and to cover all five regions. A convenience sampling method was used to select nursery staff from daycare centers in Amman, Jordan, based on their willingness to participate and accessibility. The total enrolment number of nurseries’ staff was 62 nurseries’ staff from 20 nurseries distributed in all regions of Amman.

### Data collection

The original questionnaire was adapted from a validated instrument previously developed and used in China to assess nutritional knowledge and practices among childcare providers. The translation into Arabic was carried out by a certified translation and statistical auditing office recommended by the university. The translated version was reviewed for linguistic and cultural appropriateness. Content validity was ensured through expert review, and internal consistency was assessed using Cronbach’s alpha. It included three components: socio-demographic characteristics, nutritional knowledge part, and practice’s part (18, 19).

Socio-demographic characteristics include age, with the age of the nursery staff rounded to the nearest year. Per Article 9 of the instructions of the Ministry of Social Development to license nurseries, the minimum age of caregivers is established at 20 years, so we categorized the age groups as 20–29, 30–39, and above 40. Marital status was categorized as married or single. Educational level was categorized into either in secondary school or in higher education institutions (e.g., universities or colleges). Employment position of nursery staff: manager and teacher. The socio-demographic characteristics omitted a gender question, as all nursery staff were females according to the instructions of the Ministry of Social Development.

Those who were not employed at the time of data collection or who did not give their consent to participate in the study were excluded. The inclusion criteria were: nursery staff members who were 20 years of age or older, working in licensed nurseries, and actively involved in teaching or managing.

The second section was nutritional knowledge, featuring 17 multiple-choice questions on various nutrition topics. Each question presented three choices. The response answers for this section were: yes, no, or I do not know. Each correct answer was granted with one (1) point, and the nutrition knowledge level was evaluated out of 17 total points. The degree of nutrition knowledge levels scored were categorized into three categories based on previous studies (20, 21). Scores beyond 75% (13–17 points) are classified as excellent; scores from 50 to 75% (8–12 points) are considered moderate; and below 50% (8 points and below) are deemed poor. However, at the time of the study, there was no national policy mandating compulsory nutrition training for nursery staff in Jordan. Based on self-report, the majority of participants indicated that they had not received formal training in nutrition before or during their employment at the nursery.

The final section on nutritional practices comprised 10 questions. Three statements on general nutrition and seven statements concerning job-related practices were incorporated. The responses to questions on job-related practices from numbered 18 to 24 were: yes, no, or maybe, and the responses to general nutrition practices 25 and 26 were: always, often, sometimes, or never, and question 27 was: yes, or no answer. The main scores of practices were used to classify the nutritional practice levels into three groups according to previous studies (20, 21). Scores above 8 (>75%) were considered ‘excellent’; scores of 5–7 (50–75%) were considered ‘moderate’; and scores below 5 (<50%) were considered ‘poor.’. The teachers present in nurseries participated in completing the questionnaire.

Data on participants’ nutrition-related practices were self-reported through a structured questionnaire and were not observed directly by the researchers.

### Reliability of questionnaire

The reliability of the nutritional knowledge section of 62 nursery staff in Amman, Jordan, was evaluated by assessing the internal reliability (Cronbach’s alpha was 0.75); additionally, the reliability of the practice section among 62 nurseries staff was evaluated by the internal reliability assessment (Cronbach’s alpha was 0.80). The total reliability of both components of the questionnaire included among 62 nurseries staff in Amman, Jordan, was evaluated by assessing the internal consistency (Cronbach’s alpha was 0.85).

### Statistical analysis

As the data did not follow a normal distribution, non-parametric statistical tests were used. Specifically, the Chi-square test was used to analyze the distribution of responses to knowledge and practice items, while the Spearman correlation test was used to assess the relationship between nutritional knowledge and practices.

### Results

#### Participants characteristics

This study assessed the outcomes of 20 Amman nurseries. In all, 62 nursery employees took part in the survey. The Ministry of Social Development’s guidelines for licensing nurseries required that caregivers had to be no younger than 20 years old. As a result, the category age group began at that age, and the age distribution of nurseries’ employees was adjusted to maintain a normal distribution. The distribution of the sample was as follows: 35.5% of the sample was between 30 and 39 years old, 43.5% was above 40 years old, and the remaining 20 to 29 years old. 75.8% of nurseries staff participants were married, while 24.2% were single, according to the distribution of participants by marital status. In terms of staff education, 25.8% of participants had only completed high school, while almost two-thirds (74.2%) had a university degree. Additionally, 45.2% of participants hold a managerial job, and 54.8% of participants are teachers.

#### The relationship between nutritional knowledge and socio-demographic characteristics of nursery staff

Table 1 indicates that there were no statistically significant variations in the staff’s sociodemographic characteristics (*p* value > 0.05). However, we did find that the staff members who were older than 41 had the highest scores, while the staff members who were between the ages of 20 and 29 had the lowest scores. Participants with more education had higher results than those with only a high school degree. Married staff members scored higher than single staff members. Teachers’ scores were lower than those of staff members serving as managers. Midpoint calculations using categorized data were used to estimate the overall mean knowledge score, which came out to 12.6 out of 17. This finding indicates that the nursery staff participants had a generally moderate to high level of nutritional awareness. 53.2% of the staff scored in the “excellent” category (13–17), 43.5% in the “moderate” category (8–12), and only 3.2% in the “poor” category (≤8), according to the distribution.

**Table 1:** Relationship between nutritional knowledge and socio-demographic characteristics of nurseries staff (*n* = 62).

Socio- demographic categories			Poor ≤ 8 scores	Moderate 8–12 scores	Excellent 13–17 scores	*p* value
Age (years)	20–29	Frequency (% of total)	1 (1.6%)	8 (12.9%)	6 (9.7%)	0.573
	30–39	Frequency (% of total)	0	7 (11.3%)	13 (21%)	
	≥ 40	Frequency (% of total)	1 (1.6%)	12 (19.4%)	14 (22.6%)	
Education	Secondary school	Frequency (% of total)	0	8 (12.9%)	8 (12.9)	0.624
	Higher education institutions	Frequency (% of total)	2 (3.2%)	19 (30.6%)	25 (40.3%)	
Marital status	Married	Frequency (% of total)	1 (1.6%)	21 (33.9%)	25 (40.3%)	0.676
	Single	Frequency (% of total)	1 (1.6%)	6 (9.7%)	8 (12.9%)	
Work status	Teacher	Frequency (% of total)	0	18 (29%)	16 (25.8%)	0.106
	Manager	Frequency (% of total)	2 (3.2%)	9 (14.5%)	17 (27.4%)	
Total			3.2%	43.5%	53.2%	

#### Practice of nurseries’ staff

There was a greater proportion of nursery school employees (82.3%) who expressed interest in preventing children from being forced to eat more than they desired than those who did not.

Ninety-three percent of employees sat down with children for lunch, 87.1% of employees avoided eating unhealthy food in front of children, and 74.2% of employees positively encouraged preschoolers to eat new or unfamiliar foods. Additionally, all participants (100%) urged kids to drink water throughout the day, and 96.8% of nursery staff expressed interest in discussing healthy eating with kids.

Of the staff, 11.3% maybe allowed children to consume foods high in calories and fat, such as sweets, chips, carbonated drinks, sugar-sweetened beverages, fried noodles, etc., while 14.5% (do not allow).

Over half of the employees (64.5%) reported that they always or frequently took the initiative to educate their friends or family about children’s nutrition. Among them, 29% reported always doing so, while 35.5% reported doing so frequently. Additionally, 29% reported doing so occasionally, and 6.5% stated they never took such initiative.

In their free time, 38.7% of employees frequently took the initiative to learn about children’s nutrition. To be more specific, the staff members who took the initiative to learn about children’s nutrition in their free time were always (19.4%), occasionally (37.1%), and never (4.8%). However, only 9.7% of the employees took part in training on childhood nutrition.

#### Relationship between nutritional practices of nurseries staff and socio-demographic characteristics of participants

Approximately 50% of the staff exhibited excellent nutritional practices, 38.8% demonstrated moderate practices, and 6.4% displayed poor practices, as illustrated in Table 2.

**Table 2:** Correlation between nutritional practices and socio-demographic characteristics of nurseries staff (*n* = 62).

Socio- demographic categories			Poor	Moderate	Excellent	P value
Age (years)	20–29	Frequency (% of total)	1 (1.6%)	2 (3.2%)	12 (19.4%)	0.215
	30–39	Frequency (% of total)	1(1.6%)	10 (16.1%)	9 (14.5%)	
	≥ 40	Frequency (% of total)	2 (3.2%)	12 (19.4%)	13 (21.0%)	
Education	Secondary school	Frequency (% of total)	1 (1.6%)	6 (9.7%)	9 (14.5%)	0.991
	Higher education institutions	Frequency (% of total)	3 (4.8%)	18 (29%)	25 (40.3%)	
Marital status	Married	Frequency (% of total)	3 (4.8%)	19 (30.6%)	25 (40.3%)	0.885
	Single	Frequency (% of total)	1 (1.6%)	5 (8.1%)	9 (14.5%)	
Work status	Teacher	Frequency (% of total)	2 (3.2%)	12 (19.4%)	20 (32.3%)	0.106
	Manager	Frequency (% of total)	2 (3.2%)	12 (19.4%)	14 (22.6%)	
Total			6.4%	38.8%	54.9%	

Although calculated to be significant, Table 2 did not reveal any statistically significant differences in the sociodemographic features of the staff on practices (*p* value > 0.05). We observed (liked knowing) that employees over 41 had the greatest scores, while participants between the ages of 20 and 29 had the lowest results. Higher educated staff members scored higher than individuals with only a high school diploma. Employees who were married had higher ratings than those who were single. Additionally, teachers scored higher than staff members who worked as managers. 32.3% of teachers and 22.6% of managers, respectively, earned excellent practices scores. Using the midpoint approximation, the overall mean practice score was calculated to be 7.7 out of 10. 54.9% of nursery employees had excellent practices (scoring 8–10), 38.8% had moderate practices (scores 6–7), and only 6.4% had poor practices (scores ≤5).

#### The correlation between nutritional knowledge and nutritional practices among nurseries staff

When applied to categorical data, Spearman correlation was utilized to determine a relationship between nursery staff members’ nutritional practices and knowledge. There is no correlation between knowledge and practice (*p* value ≥0.05), according to the data (p value = 0.278). Nevertheless, the high level of knowledge does not always translate into the practices.

## Discussion

The purpose of this study was to evaluate the nutritional behaviors and understanding of Amman, Jordanian nursery employees. As far the researchers are aware, this is the first study in Jordan to evaluate the nursery staff’s dietary practices and understanding. The discussion based on the analysis’s findings is presented in this chapter.

### Nutritional knowledge and practice nurseries staff questionnaire

Preschoolers’ lives are greatly impacted by the staff at the nurseries. They have the chance to help preschoolers develop better eating habits and encourage nutrition education, yet they cannot effectively support intervention and prevention plans that aim to reduce childhood obesity if they lack the appropriate nutritional knowledge (22). In order to evaluate the dietary behaviors and expertise of nursery workers in preschools, a questionnaire was developed and validated. The nutritional-related knowledge section has 17 questions. It was found that there were questions in which the percentage of the correct answers was high, and others was low. The survey’s questions about protein and fat had a lower percentage of correct answers (Q7, Q11, Q12, Q14, and Q18); this could represent a sign that nursery staff members are unaware of the sources of protein and which ones are of higher quality, as well as the significance of the fat group for kids’ health and cognitive development.

There were some discernible patterns, despite the fact that there were no statistically significant correlations between sociodemographic traits and self-reported dietary knowledge or habits (*p* > 0.05). Employees in managerial positions, for example, as well as those who were older, married, and better educated, tended to score higher. Despite not being statistically significant, these trends might represent actual differences that were missed because of statistical power constraints. Their years of expertise may help to explain this, as they have probably been exposed to more nutrition-related information and practices over time. A Chinese investigation came to a similar conclusion, as greater education gives employees a broader understanding of child development and evidence-based methods, particularly in areas linked to early childhood education and health, which enhances their overall efficacy at work (18). However, the comparatively small sample size (n = 62), which might have made it more difficult to identify statistically significant differences, is one explanation. Furthermore, the sample was selected from a small geographic area, which might restrict generalizability and increase response homogeneity. The degree and detectability of correlations between reported knowledge and behaviors and demographic characteristics were probably impacted by these factors. On the other hand, according to Macnamara and Maitra (23), greater practical knowledge, improved decision-making abilities, and a deeper comprehension of best practices are often the results of elder employees’ greater real-world experience. Experience has been demonstrated to enhance job performance in a variety of industries, such as education and healthcare. Marriage frequently promotes a greater feeling of responsibility and emotional maturity, which results in more thoughtful and conscientious work habits, especially for those who have children (24).

While teachers exceeded managers in terms of dietary practice, managers outperformed staff members in terms of nutritional knowledge. This suggests that information is not always sufficient; even if nursery staff members possess the requisite nutritional knowledge, they may not always use it in their day-to-day work. Higher knowledge might not be reflected in practices, though, as no association between knowledge and practices was found.

Barely 9.7% of the participants attended courses on children nutrition. This suggests that there is a need to hold nutrition awareness workshops for nursery staff, particularly since it is now simple to conduct these workshops via social networks (online) and require them to hold regular nutrition courses through the authorities in order to stay up to date on advancements in the field of preschool nutrition.

The percentage of staff (*N* = 62) who received correct scores in this study was 53.2%, which is comparatively low. This suggests that the nursery staff members who took part in the survey have a poor comprehension of nutritional knowledge. These results were higher than those published in a study conducted in the United States (3%) (22), and comparable to the results of a survey conducted in China, where the proportion of right answers was less than 60% (18). Public health may be greatly impacted by these findings, and research on childcare providers like nursery employees may be essential in preventing childhood obesity and advancing general health.

In conclusion, nursery employees should be educated in the fundamentals of infant nutrition, including the essential dietary needs and accepted practices for fostering normal growth and development. This information is essential for making sure that kids’ dietary needs are successfully satisfied (25). Additionally, it is crucial to have frequent training and professional development workshops that concentrate on the most recent findings and recommended practices in pediatric nutrition (26). Ongoing training guarantees that nursery employees have the knowledge and abilities needed to make wise dietary choices, promoting the general health and developmental results of the kids under their supervision.

### Study limitations

This study has several limitations. First, data were collected through convenience sampling of daycare centers, which may limit the generalizability of the findings to all daycare settings across the region. The sample may not fully represent nurseries with different staffing structures, geographic locations, or institutional policies. Another limitation is that the reported practices were self-reported by the participants through questionnaires and were not directly observed by the researchers. This may have introduced response bias, as participants might have overestimated positive behaviors or underreported less favorable practices.

## Conclusion

The purpose of this study was to evaluate the dietary habits and expertise of nursery workers and to pinpoint areas where early childhood care needs to be improved. The results showed that although nursery employees have a basic understanding of children’s nutrition, there are significant gaps that emphasize the necessity of formal training and ongoing education. These findings are consistent with our study’s goal, which was to assess existing practices and knowledge while also providing guidance for future initiatives to raise awareness among parents and caregivers and improve the nutritional value of meals served to children. In the end, this study lays the groundwork for future research on child nutrition, including evaluations of parental actions and knowledge, as well as the creation of focused interventions.

## Data Availability

The raw data supporting the conclusions of this article will be made available by the authors, without undue reservation.
